# Integration of immunogenic cell death in the treatment landscape of non-small cell lung cancer: harnessing the power of the immune system

**DOI:** 10.1038/s41420-026-03012-2

**Published:** 2026-03-18

**Authors:** Zhe Liu, Xin Xu, Meijing Wang, Jianlei Zhang, Xuesong Zhao, Meina Wang, Fang Liu, Zhonghua Liu

**Affiliations:** 1 HuaiHe Hospital, Henan University, Kaifeng, China; 2 School of Basic Medical Science, Henan University, Kaifeng, China; 3 AnYang People’s Hospital, Anyang, China

**Keywords:** Non-small-cell lung cancer, Cell death and immune response

## Abstract

Non-small cell lung cancer (NSCLC) accounts for 85% of lung cancer cases and is one of the leading causes of cancer-related death worldwide. Although traditional therapies such as chemotherapy, radiotherapy and molecular targeted therapy, as well as immunotherapy, have made substantial progress, drug resistance and tumour recurrence remain significant challenges. Immunogenic cell death (ICD), a special type of cell death, has emerged as a cutting-edge strategy for NSCLC treatment due to its unique immune activation mechanism. ICD orchestrates immunogenic tumour cell death via coordinated endoplasmic reticulum stress and reactive oxygen species generation, resulting in the release of damage-associated molecular patterns (DAMPs). Through a synergistic mechanism, tumour-associated antigens are unveiled, antigen-presenting cells such as dendritic cells are activated, and T cell responses targeting tumour-specific antigens are triggered. These factors collectively act to reprogramme the immunosuppressive tumour microenvironment (TME). Preclinical trials have demonstrated that chemotherapy, radiotherapy and molecular targeted therapy enhance the antitumour effect by inducing ICD, providing new strategies for treating NSCLC. This review systematically summarises the induction strategies of ICD in the treatment of NSCLC and focuses on the progress in preclinical experiments and clinical trials. Additionally, the current paper discusses the core challenges and future development directions of ICD in NSCLC therapy, to provide novel insights into the optimised utilisation and clinical implementation of ICD induction strategies.

## Facts

1. ICD effectively activates antitumour immune responses by releasing DAMPs such as CALR exposure, ATP secretion, and HMGB1 release, in a sequential cascade.
2. ICD is triggered by therapies via distinct mechanisms: chemotherapy primarily through endoplasmic reticulum stress; radiotherapy via DNA breakage and activation of the cGAS-STING pathway, and targeted therapy through precise interference with oncogenic signal.
3. Combining ICD-inducing treatments with immunotherapies, such as immune checkpoint inhibitors, represents a promising strategy to enhance immune activation and optimise the overall efficacy of ICD-based therapies.
4. To improve the clinical translation of ICD, future research should focus on identifying reliable predictive biomarkers and addressing immune suppression in advanced cancers to maximise therapeutic outcomes.

## Open questions

1. How do specific factors within the tumour microenvironment determine the immunomodulatory function (immune activation vs. suppression of the immune system) of particular DAMPs, such as annexin A1?
2. How can parameters such as the radiotherapy dose and fractionation regimen, and the timing of its combination with immunotherapy, be precisely optimised to maximise immunostimulatory effects and minimise inhibitory ones?
3. Do antigen-specific T cell immune responses elicited by ICD induced via different mechanisms differ qualitatively in terms of their breadth, potency, and memory formation?
4. Which clinically detectable biomarkers (or combinations thereof) can stably and specifically predict successful induction of ICD and its ultimate clinical efficacy?

## Introduction

Non-small cell lung cancer (NSCLC) is responsible for the majority of cancer-related deaths worldwide, comprising over 85% of lung cancer cases [1–3]. In 2022, there were nearly 2.5 million new diagnoses and 1.8 million deaths, representing 18.7% of the global cancer burden [4, 5]. Despite recent advances in conventional therapies such as surgery, chemotherapy and radiotherapy, the five-year survival rate for NSCLC remains low, ranging from 19 to 24% overall and less than 7% for advanced cases. Furthermore, more than 60% of patients experience recurrence or metastatic disease [6]. This challenging scenario highlights the limitations of traditional approaches in addressing tumour heterogeneity, systemic toxicity, drug resistance and the immunosuppressive tumour microenvironment [7]. To overcome these poor outcomes, immunogenic cell death (ICD) is being actively explored as a novel immunological mechanism [8].

ICD is a form of regulated cell death that is triggered by stress. It involves the release of damage-associated molecular patterns (DAMPs), such as the exposure of calreticulin (CALR), the release of adenosine triphosphate (ATP) and high mobility group box-1 protein (HMGB1). This activates dendritic cells (DCs), inducing tumour antigen-targeted T-cell responses [9–13]. ICD offers dual therapeutic action: direct tumour cytotoxicity and the induction of long-term immune memory, which impedes metastasis. Certain therapeutic approaches in clinical practice can trigger ICD, for example, specific chemotherapeutics (e.g. doxorubicin) induce the release of DAMPs via endoplasmic reticulum (ER) stress [9, 14], converting ‘cold’ tumours to ‘hot’ tumours. Radiotherapy (RT), molecular targeted therapy and photodynamic therapy (PDT) can also directly induce ICD [15–17]. Although immune checkpoint inhibitors (ICIs) cannot directly induce ICD, they can enhance ICD-triggered immune responses by relieving T-cell inhibition. This creates a synergistic effect that amplifies immune activation and therapeutic efficacy [18–20].

In light of the therapeutic potential of ICD in NSCLC, this review systematically elucidates the molecular mechanisms of ICD and critically evaluates preclinical and clinical progress in ICD-inducing therapies. By emphasising the pivotal role of ICD in remodelling the tumour microenvironment and overcoming immunosuppression, the review advances a new perspective on overcoming the limitations of conventional NSCLC treatments.

## Overview of immunogenic cell death (ICD)

ICD is characterised by the ER stress–dependent and tightly coordinated release of DAMPs. Hallmark features of this process include the early surface exposure of ‘eat-me’ signals, such as CALR and heat shock proteins (HSPs) [9, 21, 22], the regulated secretion of the chemotactic ‘find-me’ signal ATP [23], and the late passive release of the alarmin HMGB1 [24] (Table 1 provides a comparative overview of these key molecules and their roles in ICD). Together, these signals drive DCs recruitment, maturation and antigen uptake, thereby facilitating effective antigen processing and presentation. This occurs predominantly through MHC class I–restricted cross-presentation to CD8⁺ cytotoxic T lymphocytes, while MHC class II–dependent activation of CD4⁺ T cells provides supportive immune functions. Ultimately, these coordinated events give rise to a robust, tumour-specific adaptive immune response [25, 26].

**Table 1:** Comparative analysis of key ICD molecules.

Molecules	Signals	Physiological localisation	The release stage under pathological conditions	Pathological localisation	Release mechanisms	Receptors	Immune response mechanisms	Refs.
CALR	‘Eat Me’	ER	The early stage of cell apoptosis	Translocation to the plasma membrane	PI3K-regulated distal secretory pathway; PERK-mediated eIF2α phosphorylation; Caspase 8- BAP31- BAX/BAK pathway	CD91	CD91/ LRP1-dependent pathway enhances APCs debris uptake	[9, 14, 21, 22, 27–29]
HSPs	‘Eat Me’	ER	The early stage of cell apoptosis	Co-translocated with CALR to the plasma membrane surface	ER stress	CD91	CALR synergistically enhances antigen uptake and presentation capacity in APCs	[27, 28]
ATP	‘Find Me’	Mitochondria/Vesicles	The early to middle and late stages of cell apoptosis	PANX1-dependent vesicular release in apoptosis: Early stage: Active secretion (partially PANX1-dependent); Mid-late stage: Passive release (highly PANX1-dependent)	Classical secretory pathway; PERK-regulated ER stress signalling; PI3K-mediated exocytosis; Autophagy gene regulation; Caspase 3-PANX1 cleavage	P2RY2 P2RX7	P2R dual-signalling in immunity: Low [ligand]→P2RY2: Chemokine-mediated monocyte recruitment; High [ligand]→P2RX7: NLRP3 inflammasome activation	[23, 29–34]
HMGB1	‘Alarmin’	Nucleus	The final stage of cell apoptosis	Passive release post-membrane rupture	-	TLR2/4 AGER	HMGB1→TLR4/AGER→MYD88→DCs activation↑→T cell priming↑	[24, 32, 35–37]
IFN-I	Antiviral/ Antibacterial signals	-	Following viral and bacterial invasion of the host organism	Synthesised in the cytoplasm	cGAS-STING-TBK1-IRF3/NF-*κ*B pathway	IFN-α/β -receptor complex	IFNAR-driven innate immunity (macrophage/DCs/NK cells activation)	[38, 39]

The core feature of ICD is the sequential release and precise regulation of DAMPs. This process is initiated by ER stress, which drives the translocation of ER-resident chaperones, including CALR and the HSPs HSP70 and HSP90, to the cell surface, thereby generating an ‘eat-me’ signal. Surface-exposed ER chaperones subsequently promote phagocytosis by antigen-presenting cells (APCs) through engagement of low-density lipoprotein receptor-related protein 1 (LRP1/CD91) [27, 28]. Notably, although therapy-induced CALR exposure shares certain conserved features, the signalling pathways that culminate in ICD are stimulus-specific. For instance, both anthracycline-based chemotherapy and hypericin-based PDT depend on a phosphoinositide 3-kinase (PI3K)-regulated secretory pathway to facilitate CALR externalisation [29]. However, chemotherapy-induced ICD uniquely involves PERK-dependent phosphorylation of eukaryotic initiation factor 2α (eIF2α) and activation of a caspase-8–dependent pathway, whereas hypericin-based PDT triggers BAX/BAK activation through B cell receptor-associated protein 31 (BAP31), highlighting mechanistic divergence in ICD initiation across therapeutic modalities [14].

During the early stages of ICD, in addition to CALR exposure, ATP is actively secreted through PERK/PI3K-dependent secretory pathways in dying cells where plasma membrane permeabilisation has not yet occurred [29–31]. As the cell death process progresses, the integrity of the plasma membrane gradually deteriorates. Activated Caspase 3 cleaves pannexin 1 (PANX1), forming channels in the plasma membrane that facilitate ATP release partially dependent on these PANX1 channels. Following membrane permeabilisation, the ATP release mechanism shifts to rely primarily on extensive passive diffusion through these channels [23, 32]. Extracellular ATP acts as a key ‘find-me’ signal, eliciting immune responses through a concentration-dependent biphasic mechanism. For example, low concentrations (EC₅₀ < 1 μM) activate the purinergic P2Y2 receptor on APCs to recruit monocytes, while high concentrations (EC₅₀ > 100 μM) stimulate the purinergic P2X7 receptor, promoting NLRP3 inflammasome activation, interleukin-1β (IL-1β) secretion, and enhanced γδ T cell and CD8⁺ T cell immune effect [30, 33, 34].

During the terminal phase of ICD, progressive plasma membrane permeabilisation permits the passive release of HMGB1 [24]. In monocytes and macrophages, extracellular HMGB1 exerts cytokine-like activities, the magnitude and context of which are regulated by inflammatory cues such as tumour necrosis factor (TNF), lipopolysaccharide and IL-1β [32]. The immunostimulatory function of HMGB1 is mediated through its interaction with pattern recognition receptors (PRRs), most notably Toll-like receptor 2 (TLR2) and Toll-like receptor 4 (TLR4) [35]. Among these pathways, activation of the HMGB1–TLR4–MYD88 signalling axis is particularly critical for the initiation of adaptive immune responses. Engagement of this pathway enhances antigen processing and presentation efficiency in DCs, thereby reinforcing innate immune co-stimulatory signals and ultimately promoting robust antitumour adaptive immunity [36, 37].

In addition to the aforementioned classic DAMPs, other signalling molecules such as type I interferons (IFN-I) and annexin A1 (ANXA1) also play an important and complex role in ICD. IFN-I are essential innate immune mediators that are triggered by cytosolic DNA during infection. DNA binds to cyclic GMP-AMP synthase (cGAS), catalysing the formation of the second messenger cyclic GMP-AMP (cGAMP). This activates the ER protein stimulator of interferon genes (STING). STING then recruits and phosphorylates TANK-binding kinase 1, which leads to the phosphorylation of nuclear factor kappa-B (NF-*κ*B). This ultimately drives the expression of IFN-I and cytokine [38]. Secreted IFN-I then signals through the IFN-α/β receptor, inducing an antiviral/antibacterial state in neighbouring cells and promoting the activation of macrophages, DCs, and natural killer (NK) cells, thereby enhancing overall immune defence [39]. ANXA1 can limit inflammatory spread or enhance chemotherapy-induced ICD by binding to the formyl peptide receptors FPR2 or FPR1, respectively. The latter process potentially involves the activation of PRRs, such as TLR3/TLR4 [40]. Notably, ANXA1 exhibits context-dependent immunomodulation. In NSCLC, it binds to poly ADP-ribose polymerase 1 and upregulates *Stat3*-mediated PD-L1, thereby promoting immune evasion, proliferation, and metastasis [41]. Therefore, in NSCLC, ANXA1 may not act as an ICD-associated danger signal, but it could potentially be targeted to modulate tumour progression.

In summary, ICD links tumour cell death to antitumour immunity by orchestrating the release of DAMPs. Its core mechanism involves the exposure of CALR and the release of ATP and HMGB1, which collectively activate innate and adaptive immune responses, remodel the TME, reverse immune suppression and amplify antitumour effects. Future efforts should clarify how tumour heterogeneity affects ICD induction, identify biomarkers to predict ICD efficacy, and develop combination strategies based on the underlying mechanisms to enable precise, clinically translatable ICD induction.

## Research progress of ICD in NSCLC

It has been demonstrated that chemotherapy, RT, molecular targeted therapy and PDT can induce ICD in NSCLC, thereby activating systemic antitumour immunity. This section provides a comprehensive review of the mechanisms, clinical efficacy, and future directions of these methods in inducing ICD in the context of NSCLC.

### Chemotherapy-induced ICD

Conventional chemotherapeutic agents such as anthracyclines, taxanes, and platinum-based drugs can induce ICD and activate antitumour immunity in NSCLC. However, their non-selective cytotoxicity not only kills tumour cells but also damages immune cells and induces systemic toxicity. By contrast, targeted chemotherapy, as exemplified by antibody-drug conjugates (ADCs), enables the precise delivery of cytotoxic payloads and the induction of ICD, offering a key strategy to mitigate the systemic toxicity of conventional chemotherapy and achieve precise immune modulation. This section systematically elucidates the similarities and differences in ICD induction by these agents, evaluates the efficacy and safety of their clinical translation, and considers future research directions.

**Fig. 1:**
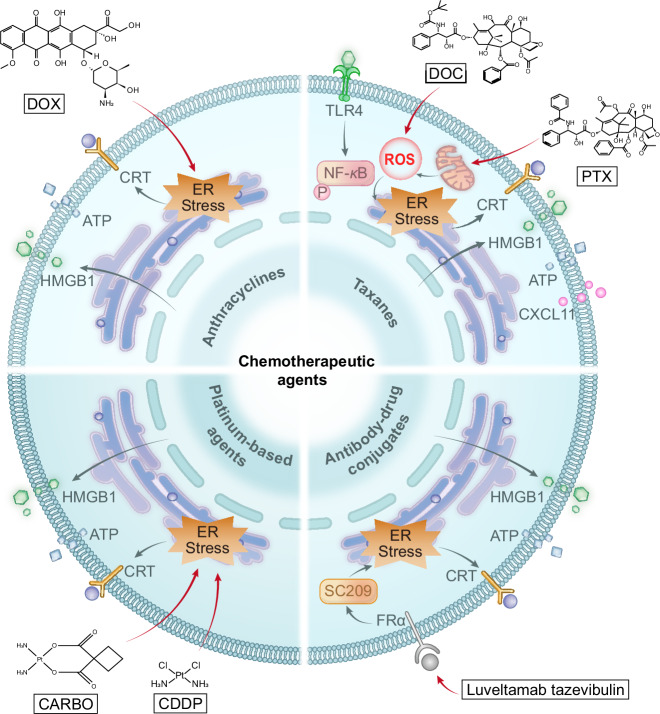
The commonalities and differences in the mechanism of chemotherapeutics induced ICD in NSCLC. Anthracyclines such as DOX, taxanes such as PTX and DOC, platinum-based agents such as CDDP and CARBO, ADCs such as luveltamab tazevibulin, could trigger ICD through CALR exposure, ATP secretion, and HMGB1 release. Among which, DOC increases HMGB1 release in a ROS-dependent manner, thereby activating the NF-*κ*B pathway.

In the treatment of NSCLC, certain anthracyclines, taxanes and platinum-based agents can induce ICD via various mechanisms and with varying potency (Fig. 1). For example, anthracyclines such as doxorubicin (DOX) trigger the release of ATP and HMGB1 and the exposure of CALR, thereby activating T cells and M1 macrophage polarisation. This results in a time-lagged ICD effect [42–44]. Among taxanes, paclitaxel (PTX) promotes the release of DAMPs via mitochondrial oxidative stress, enhancing DCs maturation and T/NK cell proliferation [45, 46]. Docetaxel (DOC) increases HMGB1 release in a reactive oxygen species (ROS) -dependent manner, activating the NF-*κ*B pathway and synergising with platinum drugs to enhance ICD [47, 48]. Among platinum-based agents, cisplatin (CDDP) alone induces the highest levels of DAMPs, while carboplatin (CARBO) has weaker effects, but enhances ATP release and CALR exposure when combined with taxanes. This results in stronger immune activation and antitumour responses in both vitro and in vivo settings [45, 48]. Future investigations should optimise combination strategies according to the heterogeneity of the ICD and clarify the underlying synergistic mechanisms.

In clinical practice, chemotherapeutic agents that induce ICD exhibit distinct efficacy and toxicity profiles (see Table 2 for more clinical data). Li et al. reported that, compared to inhalable CDDP, inhalable DOX demonstrated superior reduction in cancer nodule size (5.88% ± 3.98% vs. 4.15% ± 2.92%) and lower haematological and alopecia toxicity (e.g. anaemia 25% vs. 45%, alopecia 1% vs. 10%) [49]. Schiller et al. found that combinations such as PTX plus CDDP/CARBO and DOC plus CDDP had a comparable objective response rate (ORR) of 17–21% and median survival of 7.4–8.1 months in advanced NSCLC. However, they differed significantly in terms of toxicity: PTX + CDDP was associated with more pronounced haematological toxicity (e.g. a 75% risk of grade 3–4 neutropenia) and gastrointestinal reactions. In contrast, PTX + CARBO presented a lower risk of gastrointestinal toxicity and febrile neutropenia. For DOC + CDDP, hypersensitivity reactions require attention, with a 7% risk of grade 3–4 events [50]. Furthermore, Belani et al. observed an ORR of 43% (95% CI: 24–63%) and a median overall survival (OS) of 13.9 months (range 1–35+ months) with DOC + CARBO, albeit with notable haematological toxicity (a 79% risk of grade 4 neutropenia) [51]. Therefore, treatment selection should be individualised based on these distinct toxicity profiles.

**Table 2:** ICD-related clinical research data.

Therapy	Study identifier (acronym)	Phase	NSCLC setting (accrual)	Therapeutic regimen	mOS (95% CI) | month	ORR | %	mPFS (95% CI) | month	sAE | %
Chemotherapy	NCT02358473	Ⅰ	Stage IIIB–IV (*N* = 13)	Mogamulizumab + Docetaxel	8.88 (2.70– 8.88)	-	1.87 (0.76– 4.47)	46.15
	NCT05553808	Ⅱ	Measurable (*N* = 105)	Docetaxel	8.2 (4.5–16.1)	3.3	4.8 (2.8–NA)	47.06
				Feladilimab + Docetaxel	7.8 (4.7–10.8)	3.4	4.3 (2.4–8.7)	48.57
	NCT04880863	ⅡA	Stage Ⅳ (*N* = 38)	Obinutuzumab > NAP + Docetaxel	8.8 (6.4–11.7)	8.8	12.2 (1.6– 17.7)	42.11
	NCT02642042	Ⅱ	Stage IV or recurrent, KRAS mutation positive (*N* = 54)	Trametinib + Docetaxel	10.9 (8.0–14.0)	-	4.1 (3.1–5.1)	57.41
	NCT03228186	Ⅱ	Stage Ⅳ or recurrent (*N* = 40)	Pevonedistat + Docetaxel	13.2 (7.4–NA)	-	4.1 (2.9–7.0)	40.00
	NCT04364620	Ⅱ	Stage III–IV, measurable (*N* = 35)	AB-16B5 + Docetaxel	7.8 (4.9–14.6)	17.1	3.9 (1.6–7.7)	65.71
	NCT00271505	Ⅱ	Stage Ⅳ (*N* = 40)	Carboplatin + Docetaxel + Bevacizumab	16.5 (13.6– 31.2)	-	7.9 (6.0– 11.1)	32.50
	NCT02387216	Ⅱ	Stage Ⅲ, Heregulin Positive or Stage Ⅳ (*N* = 152)	MM-121 + Docetaxel	7.7 (3.6–10.4)	19.7	3.4 (1.9–5.7)	38.83
				Docetaxel	8.4 (5.8–14.7)	5.3	4.1 (2.7–6.3)	30.61
	NCT01702844	Ⅱ	Stage Ⅳ (*N* = 42)	Nab Paclitaxel	9.3 (6.4–12.1)	-	5.2 (2.0–7.4)	35.71
	NCT02367794	Ⅲ	Stage Ⅳ (*N* = 1021)	Nab-Paclitaxel + Carboplatin	13.5 (12.2– 15.1)	41.0	5.6 (5.5–5.7)	28.74
				Atezolizumab + Nab-Paclitaxel + Carboplatin	14.2 (12.3–16.8)	49.7	6.5 (5.7–7.1)	50.30
				Atezolizumab + Paclitaxel + Carboplatin	12.6 (11.6–14.7)	49.3	5.6 (5.5–6.9)	45.48
	NCT03594747	Ⅲ	Stage Ⅳ, Untreated (*N* = 360)	Tislelizumab + Paclitaxel + Carboplatin	26.1 (18.99–33.81)	70.0	10.6 (7.03– 16.23)	46.67
				Tislelizumab + Nab-paclitaxel + Carboplatin	23.3 (18.76– 26.38)	9.8	8.8 (8.05– 11.93)	46.61
				Paclitaxel + Carboplatin	19.4 (15.97– 23.43)	5.5	4.8 (2.86– 6.11)	24.70
	NCT01285609	Ⅲ	Stage Ⅳ or Recurrent (*N* = 749)	Ipilimumab + Paclitaxel + Carboplatin	10.94 (9.56– 12.02)	-	5.55 (5.36– 5.85)	63.16
				Placebo + Paclitaxel + Carboplatin	10.74 (9.66–11.73)	-	5.59 (5.52– 5.72)	49.68
	NCT02250326	Ⅱ	Stage Ⅳ (*N* = 240)	Nab-Paclitaxel + CC-486	8.1 (6.64–11.86)	-	3.2 (2.30– 4.30)	37.97
				Nab-Paclitaxel + Durvalumab	10.1 (7.75– NA)	-	4.5 (3.45– 5.88)	56.41
				Nab-Paclitaxel	17.0 (8.21– NA)	-	4.2 (2.79– 5.06)	37.97
	NCT00807612	ⅠB/Ⅱ	Stage Ⅳ (*N* = 15)	Ganitumab + Paclitaxel / Carboplatin	-	27.0	4.6 (3.2–5.4)	50.00
	NCT00795340	Ⅲ	Stage ⅢB–Ⅳ (*N* = 306)	Carboplatin + Cediranib maleate + Paclitaxel	12.2 (9.1–18.0)	-	5.52 (4.86– 6.05)	43.79
				Carboplatin + Placebo + Paclitaxel	12.1 (11.1– 15.5)	-	5.45 (4.86– 5.78)	35.29
	NCT03128008	Ⅱ	Stage II–IIIB, Unresectable (*N* = 10)	Carboplatin + Paclitaxel + RT (60Gy/6fx)	NA (NA–NA)	-	13.39 (2.58–NA)	60.00
	NCT02328105	Ⅱ	Stage Ⅳ (*N* = 11)	Carboplatin + Abraxane	30 (2.2–NA)	-	7.3 (2.2–13)	18.18
	NCT02657434	Ⅲ	Stage IV, non-squamous (*N* = 578)	Carboplatin / Cisplatin + Pemetrexed	13.6 (11.0– 15.7)	37.4	5.2 (4.3–5.6)	33.21
				Atezolizumab + Carboplatin / Cisplatin + Pemetrexed	17.5 (13.2– 19.6)	51.7	7.7 (6.7–8.5)	51.20
	NCT00948675	Ⅲ	Stage IV, non-squamous (*N* = 361)	Pemetrexed + Carboplatin	10.51 (9.26– 11.96)	-	4.44 (4.21– 5.32)	44.44
				Paclitaxel + Carboplatin + Bevacizumab	11.66 (9.17– 14.32)	-	5.45 (5.03– 5.95)	38.55
	NCT00741988	Ⅱ	Stage III–IV, Unresectable (*N* = 82)	Ixabepilone + Carboplatin	9.3 (6.4–16.6)	-	5.3 (2.8–8.6)	33.33
				Ixabepilone + Carboplatin + Bevacizumab	13.1 (8.9–NA)	-	6.7 (5.1–8.4)	45.00
	NCT03965689	Ⅱ	Stage IIIB–Ⅳ (*N* = 27)	Paclitaxel + Carboplatin + Pevonedistat	7.73 (0.79–21.71)	-	3.68 (0.46–19.67)	64.00
	NCT00982111	Ⅲ	Stage IV, nonsquamous (*N* = 633)	Necitumumab + Pemetrexed + Cisplatin	11.3 (9.5–13.4)	31.1	5.6 (5.1–6.0)	51.97
				Pemetrexed + Cisplatin	11.5 (10.1–13.1)	32.1	5.6 (4.8–5.7)	41.67
	NCT00981058	Ⅲ	Stage IV, squamous (*N* = 1093)	Necitumumab + Gemcitabine + Cisplatin	11.5 (10.4–12.6)	31.2	5.7 (5.6–6.0)	48.70
				Gemcitabine + Cisplatin	9.9 (8.9–11.1)	28.8	5.5 (4.8–5.6)	38.45
	NCT01544179	Ⅲ	Stage III–IV, EGFR mutation positive (*N* = 265)	Gefitinib + Cisplatin + Pemetrexed	14.8 (10.4–19.0)	31.6	5.4 (4.5–5.7)	28.79
				Placebo + Cisplatin + Pemetrexed	17.2 (15.6–NA)	34.1	5.4 (4.6–5.5)	21.21
**Antibody drug conjugates (ADCs)**	NCT05513703	Ⅱ	Stage Ⅲ–Ⅳ, non-squamous (*N* = 9)	Telisotuzumab Vedotin	17.5 (0.9–NA)	33.3	4.0 (0.9–NA)	33.33
	NCT04394624	-	Stage IV, Non-squamous, CEACAM5-positive (*N* = 31)	Tusamitamab Ravtansine+ Ramucirumab	-	22.6	5.95 (5.355– 9.101)	22.58
	NCT05245071	Ⅱ	Nonsquamous (*N* = 22)	Tusamitamab Ravtansine	-	9.1	1.87 (1.676– 3.614)	31.82
	NCT04619004	Ⅱ	Stage Ⅲ–Ⅳ, EGFR-mutated (*N* = 227)	Patritumab Deruxtecan 5.6 mg/kg	-	28.4	5.5 (5.1–5.9)	40.00
				Patritumab Deruxtecan Up-Titration	-	16.0	6.7 (4.2–8.8)	32.00
	NCT05246514	Ⅱ	Stage Ⅳ, HER2-mutated (*N* = 172)	Trastuzumab Deruxtecan	NA (NA–NA)	58.3	NA (7.2–NA)	45.83
	NCT04644237	Ⅱ	Stage Ⅳ, HER2-mutated (*N* = 152)	Trastuzumab Deruxtecan 5.4 mg/kg	19.0 (14.7–NA)	50.0	9.7 (8.2–11.3)	41.58
				Trastuzumab Deruxtecan 6.4 mg/kg	17.9 (13.8– NA)	56.0	9.5 (6.5–15.1)	52.00
**Radiotherapy**	NCT03812549	Ⅰ	Stage IV, PD-L1 Positive (*N* = 29)	Sintilimab + SBRT (30Gy/3fx) + LDRT (2Gy/1fx)	NA (NA– NA)	66.7	7.228 (0– 17.323)	0.00
				Sintilimab + SBRT (30Gy/3fx) + LDRT (4Gy/2fx)	NA (NA– NA)	62.5	9.035 (1.122– 16.948)	29.41
				Sintilimab + SBRT (30Gy/3fx) + LDRT (10Gy/5fx)	15.639(0–32.8)	50.0	4.501 (0– 12.861)	16.67
	NCT02658097	Ⅱ	Stage IV (*N* = 13)	Pembrolizumab + SFRT (8Gy)	9.2 (2.6–NA)	-	3.5 (1.4–NA)	38.46
	NCT05691829	Ⅱ	Stage IV, untreated (*N* = 14)	Pembrolizumab + Chemotherapy + RT (20Gy/5fx)	11.91 (1.49–18.67)	38.46	9.90 (1.49– 18.67)	64.29
	NCT00301808	Ⅱ	Stage Ⅲ (*N* = 28)	Cisplatin + Docetaxel + Pemetrexed disodium + RT	33.7 (6.4–NA)	-	16.9 (7.2–36.2)	51.72
	NCT02045446	Ⅱ	Stage Ⅳ, have received first line chemotherapy (*N* = 29)	Chemotherapy	6.3 (0.9–19.6)	-	3.5 (3.387– 4.315)	40.00
				Chemotherapy + SBRT	6.8 (0.2–22.6)	-	9.7 (9.587– 10.515)	57.14
	NCT02314364	Ⅱ	Stage IV, Oncogene-driven (*N* = 27)	SBRT > TKI	70.8 (41.8–NA)	-	14.7 (8.3–46.4)	0.00
	NCT00547105	Ⅱ	Stage Ⅲ–IV (*N* = 24)	Erlotinib+SBRT	20.4 (3–60)	-	14.7 (2–60)	20.83
**Molecular targeted therapy**	NCT01945021	Ⅱ	Stage Ⅲ, ALK negative, ROS1 positive (*N* = 127)	Crizotinib	44.2 (32.0–NA)	69.3	15.9 (12.9– 24.0)	36.22
	NCT01639001	Ⅲ	Stage Ⅲ, ALK positive (*N* = 207)	Crizotinib	33.7 (26.5–42.5)	87.5	11.1 (8.3–12.6)	44.23
				Chemotherapy	32.9 (23.9–43.1)	45.6	6.8 (5.7–7.0)	12.87
	NCT03414814	Ⅱ	EGFR Exon 20 insertion mutation (*N* = 15)	Osimertinib	6.5 (3.9–29)	0.0	3.8 (1.7–5.5)	53.33
	NCT02442349	Ⅱ	Stage IIIB–IV, T790M mutation (*N* = 171)	Osimertinib	23.2 (20.0–26.7)	62.0	9.7 (7.0–11.1)	24.56
	NCT02841579	IIa	Stage Ⅲ–Ⅳ, non-squamous (*N* = 22)	Osimertinib	28.39 (25.62– NA)	77.3	23.1 (14.1– NA)	45.45
	NCT02094261	Ⅱ	Stage Ⅲ–Ⅳ, EGFR and T790M mutation positive (*N* = 210)	Osimertinib	8.6 (8.28–9.72)	70.9	-	20.00
	NCT05215951	Ⅱ	Stage IIIB-IVB, uncommon EGFR mutations (*N* = 4)	Osimertinib + Standard Chemotherapy	NA (3.94–NA)	75.0	NA (3.29–NA)	0.00
	NCT03133546	Ⅱ	Stage IIIb–Ⅳb, confirmed EGFRm and T790M (*N* = 155)	Osimertinib + Bevacizumab	24.0 (17.8–32.1)	55.1	15.4 (9.2–18)	43.42
				Osimertinib	24.3 (16.9–37.0)	54.5	12.3 (6.2–17.2)	35.06
	NCT00376623	Ⅱ	Stage IIIB–IV (*N* = 95)	BI 2536 (200mg/Day1)	7.9 (6.0–12.3)	-	1.6 (1.5–2.8)	41.67
				BI 2536 (50mg/Day 1 - Day 3)	8.1 (6.3–13.0)	-	1.7 (1.4–3.0)	30.77
				BI 2536 (60mg/Day 1 - Day 3)	6.0 (3.4–8.1)	-	1.6 (1.4–3.2)	57.14

Single chemotherapeutic agents often cause severe systemic toxicity due to their limited selectivity. In contrast, ADCs deliver cytotoxic payloads directly to tumour cells via antibody-mediated targeting. This allows for the potent localised killing of cells and the induction of ICD, while minimising toxicity to healthy tissues. Preclinical studies have shown that ADCs such as luveltamab tazevibulin (Fig. 1), 1959-sss/DM4 and datopotamab deruxtecan trigger the release of DAMPs (e.g. HMGB1, ATP and CALR), thereby activating antitumour immunity (see Table 3 for more details and examples) [52–54]. In clinical trials (see Table 2 for more clinical data), tusamitamab ravtansine (DM4 payload) achieved an ORR of 20.3% with CEACAM5-high non-squamous NSCLC [55]. Datopotamab deruxtecan has demonstrated significant efficacy in the treatment of advanced/metastatic NSCLC, achieving an ORR of 35.8% (95% CI: 27.8–44.4) in the TROPION-Lung05 trial. Furthermore, it has shown superior median progression-free survival (PFS) compared to DOC (4.4 vs. 3.7 months; HR: 0.75, *p* = 0.004) in TROPION-Lung01 trial. Additionally, it has been associated with a lower incidence of grade ≥3 treatment-related adverse events (25.6% vs. 42.1%). However, manageable risks such as adjudicated drug-related interstitial lung disease/pneumonitis (3.6–8.8%) require monitoring [56, 57]. Thus, ADCs represent a promising strategy for balancing efficacy and safety in NSCLC by inducing targeted ICD.

**Table 3:** Differences in ICD induced by specific therapies.

Therapy	Drugs	Treatment mechanism	ICD signals	Significance	Ref
Chemotherapy	DOX	DOX induces ICD to initiate immune response, triggers specific T-cell immunity, promotes Th1 cell differentiation, and induces macrophage polarisation to M1 phenotype.	CALR, ATP, HMGB1	DOX primes tumour-specific T-cell immunity, skews Th1 polarisation, and drives macrophage M1 polarisation, while exhibiting markedly lower haematologic and non-haematologic toxicity than inhaled cisplatin, thus offering a low-toxicity chemotherapeutic option for NSCLC.	[43]
	PTX	PTX elicits ICD via mitochondrial oxidative stress, triggering DAMPs release, potentiating dendritic-cell maturation, and driving expansion of T and NK cells.	CALR, ATP, HMGB1	PTX enhances dendritic-cell maturation and expands both T and NK compartments; when combined with platinum agents it amplifies ICD, offering an effective therapeutic regimen for advanced NSCLC.	[46]
	DOC	DOC dose-dependently boosts HMGB1 release via ROS, triggers NF-κB phosphorylation, up-regulates CXCL11, and enhances HER2-CART infiltration; combination with platinum synergistically intensifies ICD.	HMGB1	DOC provides a highly effective first-line combination regimen for treatment-naïve NSCLC patients, with established efficacy when combined with platinum-based therapy.	[47]
	CDDP	CDDP alone elicits the highest level of DAMPs release and, when combined with taxanes, synergistically potentiates anti-tumour efficacy.	CALR, ATP, HMGB1	CDDP remains the backbone of NSCLC chemotherapy; its combination with paclitaxel is a proven first-line regimen for advanced disease. Despite appreciable toxicity, the robust anti-tumour activity establishes a critical platform for subsequent combination strategies.	[45]
	CARBO	Carboplatin alone elicits only modest ICD; combined with paclitaxel it markedly increases ATP secretion and CALR exposure, synergistically amplifying ICD.	CALR, ATP	The combination of CARBO and DOC can further enhance therapeutic efficacy, providing a highly effective combinatorial strategy for the clinical chemoimmunotherapy of NSCLC.	[48]
Antibody drug conjugates (ADCs)	Luveltamab tazevibulin	Luveltamab tazevibulin selectively delivers a microtubule-inhibitor payload to FRα-positive NSCLC cells, elicits ICD, and reactivates the immune compartment by enhancing CD8⁺ T-cell cytotoxicity.	CALR, ATP, HMGB1	Luveltamab tazevibulin establishes long-lived protective immunity while potentiating CD8⁺ T-cell activity, enabling precision-targeted chemotherapy and offering a novel immuno-chemotherapeutic strategy for FRα-positive NSCLC.	[52]
	1959-sss/DM4	1959-sss/DM4 selectively targets LGALS3BP⁺ neuroblastoma cells, delivers the microtubule inhibitor DM4, and elicits ICD-associated markers while enhancing intratumoural lymphocyte infiltration.	CALR, HSP70/90	1959-sss/DM4 augments intratumoural lymphocyte infiltration and exhibits substantial immune-synergistic potential.	[53]
	Datopotamab deruxtecan	Datopotamab deruxtecan targets TROP2⁺ cells, delivers topoisomerase I inhibitor DXd, and exerts anti-tumour immune effect relying on ICD.	CALR, ATP, HMGB1	The ICD immune activation mechanism of Datopotamab deruxtecan provides crucial evidence for explaining its clinical efficacy.	[54]
Radiotherapy	Bio-MnO2 NPs (a biomineralized manganese oxide nanoparticles)	Bio-MnO2 NPs combined with low-dose radiotherapy can not only sensitise radiotherapy-induced ICD by promoting the production of ROS, but also the released Mn²⁺ can activate the cGAS-STING pathway and promote CTL infiltration.	CALR, ATP, HMGB1	This approach enhances radiotherapy-induced ICD, relieves tumour hypoxia, and sparks anti-tumour immunity, offering a concise strategy for NSCLC radio-nano combination therapy.	[15]
	ZnO-Au @ mSiO_2_ (a radio-immuno-enhancer)	The ZnO-Au@mSiO₂ exhibits both radio-sensitising and immune-activating functions. Combined with low-dose X-ray, it reverses T cell exhaustion through multi-target effects such as inducing ICD, blocking the PD-1/PD-L1 pathway, and activating the cGAS-STING pathway.	CALR, HMGB1	This multi-pronged strategy synergises radiotherapy with immunotherapy by overcoming RT-induced immunosuppression through targeted ICD induction, immune checkpoint blockade, and STING activation, advancing optimised RT-ICD clinical translation for NSCLC.	[64]
Molecular targeted therapy	Crizotinib	Crizotinib, when combined with non-ICD-inducing chemotherapeutics, can trigger ICD in a T-cell and IFN‑γ‑dependent manner and concurrently up-regulate PD-1 and PD-L1 expression.	CALR, ATP, HMGB1	Crizotinib, the cornerstone of targeted therapy for ALK‑positive NSCLC, induces ICD and PD-L1 up-regulation at high dose or with chemotherapy, rationalising sequential PD-1/PD-L1 blockade.	[73]
	Ceritinib	Ceritinib elicits ICD-associated events, thereby heightening tumour immunogenicity.	CALR, ATP, HMGB1	Clinical trials have established ceritinib’s superior efficacy over first-generation ALK inhibitors, furnishing both mechanistic rationale and clinical evidence for its integration into immunocombination regimens for ALK-positive NSCLC.	[74]
	Osimertinib	Osimertinib elicits systemic anti-tumour immunity by inducing CALR exposure across diverse EGFR-mutant cell lines.	CALR	Osimertinib has established the standard-of-care across all stages of EGFR-mutant NSCLC; its capacity to mobilise systemic anti-tumour immunity via ICD induction offers a mechanistic framework that broadens the scientific rationale for its pervasive clinical benefit.	[17]
	Bemcentinib	Bemcentinib, alone or in combination, robustly elicits canonical ICD signatures and modestly up-regulates MHC-I, thereby potentiating tumour immunogenicity.	CALR, ATP, HMGB1	The ICD-inducing capacity of bemcentinib offers a novel mechanistic perspective for integrating immunotherapy throughout the entire disease trajectory of EGFR-mutant NSCLC.	[75]
	Marsdenia tenacissima extract	Marsdenia tenacissima extract suppresses AXL phosphorylation, thereby provoking ER stress that precipitates mitochondrial membrane-potential collapse, ROS accumulation, and the release of ICD-associated molecules.	CALR, ATP, HMGB1	Co-administration of Marsdenia tenacissima extract with chemotherapy enhances efficacy while attenuating toxicity, furnishing both mechanistic rationale and clinical evidence for integrating natural agents into NSCLC therapeutics.	[76]
	anlotinib	Anlotinib induces ICD to enhance dendritic-cell phagocytosis and maturation, while preventing AKT/mTORC1- and Pparδ-mediated M2 polarisation of Tumour-associated macrophages; this immune-activating effect is further amplified when combined with PD-1 inhibitors.	CALR, ATP, HMGB1	Anlotinib reshapes the tumour immune microenvironment through ICD, providing a mechanistic rationale for its combination with ICIs.	[77]
	BI2536	By targeting PLK1, BI2536 disrupts Tumour-cell mitosis and elicits ICD, thereby indirectly activating DCs, promoting T-cell priming and infiltration, while concomitantly down-regulating immune-checkpoint molecules to reprogramme the tumour immune microenvironment.	CALR, ATP, HMGB1	BI2536 uncovers a novel immunomodulatory role for PLK1 inhibitors, redirecting NSCLC therapy; as an ICD inducer it can be partnered with immunotherapeutics to overcome acquired resistance to immune-checkpoint blockade.	[87]
	STK11@PPCM	STK11@PPCM restores STK11 expression in NSCLC, reinstates ICD to drive dendritic-cell maturation, amplifies CD8⁺ T cells and M1 macrophages, and concomitantly up-regulates PD-L1, sensitising STK11-mutant tumours to αPD-1 blockade.	CALR, ATP, HMGB1	STK11@PPCM offers a targeted-delivery strategy for STK11-mutant NSCLC—an immunotherapy-refractory subset—markedly sensitising tumours to anti-PD-1 therapy and providing a readily translatable clinical regimen.	[88]
Photodynamic therapy	MB-PDT	MB-PDT induces ICD in NSCLC by triggering ER stress-driven CALR surface exposure and enhancing ICAM-1 transcription/protein synthesis.	CALR	This therapy simultaneously suppresses tumour proliferation via Bcl-2 downregulation/Caspase-3/7 activation and enhances CD8⁺ T-cell-mediated tumour lysis through granzyme B, synergising direct cytotoxicity with anti-tumour immunity.	[91]
	PMIC-NC	PMIC-NC acts as a mitochondria-targeted ROS supergenerator that induces hypoxia-enhanced Type I ROS bursts via proton transients and triggers dual Type I/II ROS under NIR irradiation through electron/energy transfer, eliciting robust ICD.	ATP, HMGB1, CALR	NIR-triggered PDT, hypoxia-boosted chemotherapy, and ICD induction co-localised to mitochondria/lung yield a single-agent immunogenic photochemical therapy that eradicates primary, distant, and metastatic NSCLC.	[16]
	ICG@MnO_2_@Exo-anti-PD-L1	The ICG@MnO₂@Exo-anti-PD-L1 nanoplatform reverses immunosuppression by blocking PD-L1, generating O₂ via MnO₂-catalysed H₂O₂ decomposition, and activating STING (via Mn²⁺)/NF-*κ*B pathways to drive M1 macrophage polarisation and ICD induction.	HMGB1	This multitargeted strategy synergises checkpoint blockade, oxygenation, innate immune activation, and macrophage repolarization to overcome immunosuppression and potentiate anti-tumour immunity through robust ICD.	[92]
	BPQDs@Lipo-YSA	BPQDs@Lipo-YSA targets NSCLC to induce NIR-triggered mitophagy via the PRKN/AKT1 pathway, thereby eliciting potent ICD.	CALR, HMGB1	This NIR-responsive ICD strategy significantly enhances tumour cell cytotoxicity in vitro and suppresses tumour growth/survival in vivo.	[93]
Oncolytic viruses	Coxsackievirus B3	Coxsackievirus B3 selectively infects NSCLC cells via CAR/DAF receptors, induces caspase-mediated apoptosis, modulates PI3K/Akt and MEK/ERK pathways for viral replication, and triggers ICD signals to synergise direct oncolysis with anti-tumour immunity for systemic effects.	CALR, ATP, HMGB1	A well-tolerated oncolytic agent, Coxsackievirus B3 achieves complete regression of therapy-refractory NSCLC xenografts by integrating direct oncolysis with ICD-induced immune activation, offering promise for localised and metastatic NSCLC treatment.	[99]
	Coxsackievirus A11	CVA11 selectively infects NSCLC cells via ICAM-1, induces caspase-mediated apoptosis (cleaved PARP detected), and exerts dose/time-dependent cytotoxicity, synergising direct oncolysis with ICD-induced immunity.	CALR, HMGB1	As a well-tolerated oncolytic virus, it achieves complete regression of NSCLC xenografts (including EGFR-mutant refractory tumours) and holds promise for combination with immunotherapy.	[100]
	Recombinant vaccinia virus	Recombinant vaccinia virus selectively replicates in NSCLC cells with constitutive ERK activation, triggers ICD, and synergises with tepotinib to enhance CD4⁺/CD8⁺ T-cell infiltration, integrating direct oncolysis with ICD-induced anti-tumour immunity to inhibit both local and distant tumours.	ATP, HMGB1	A safe and effective oncolytic agent, it converts "cold tumours" to "hot tumours" via ICD, achieves superior anti-tumour effects versus monotherapy, benefits metastatic NSCLC, and provides a promising strategy for combining with immunotherapy/targeted therapy.	[101]
	Oncolytic herpes simplex virus	Oncolytic herpes simplex virus invades NSCLC cells via nectin-1, induces ICD, and synergises with scFvPD-1 to activate anti-tumour immunity, enhance T-cell infiltration, and sensitise tumours to cisplatin.	ATP	Reverses immunosuppression in refractory NSCLC metastases, boosts PD-1 inhibition/chemotherapy efficacy, and offers a promising locoregional strategy for leptomeningeal brain metastasis.	[102]
Tumour treating fields (TT Fields)		TTFields induce ICD in NSCLC cells, activate Stat1/Irf1 signalling, upregulate CCL2/8-CCR2 and CXCL9/10-CXCR3 axes, enhance CD4⁺/CD8⁺ T-cell infiltration, and synergise with anti-PD-1 therapy to suppress tumour growth.	ATP, HMGB1	As a low-toxicity physical therapy, it reverses the immunosuppressive tumour microenvironment of NSCLC, potentiates anti-PD-1 efficacy (approved by FDA), provides a novel combination strategy for PD-L1-low/negative NSCLC, and identifies CCL2/8/CXCL9/CXCL10 as potential biomarkers.	[103]

In summary, chemotherapeutic agents, including DOX, PTX, DOC, CDDP, and CARBO, can induce ICD in NSCLC through heterogeneous mechanisms. While combination regimens of these agents show comparable efficacy but distinct toxicity profiles, informing personalised selection, ADCs enable precise chemotherapy by targeting ICD induction and demonstrate promising clinical potential. Further studies are needed to elucidate the mechanistic differences in chemotherapy-induced ICD, explore timing-based combinations, and optimise ADCs application, with the aim of advancing more effective and safer individualised therapies.

### Radiotherapy (RT)-induced ICD

RT represents a classical modality capable of inducing features of ICD through the generation of DNA damage, ER stress and ROS, thereby promoting the exposure or release of DAMPs, including CALR, ATP and HMGB1. The capacity of radiotherapy to induce ICD is widely regarded as a key immunological basis underlying the so-called abscopal effect [58]. Clinically, this phenomenon refers to the regression of distant, non-irradiated tumour lesions following local irradiation of a primary site, reflecting a systemic antitumour immune response elicited by radiotherapy-induced immune activation [59–62]. Notably, this immunomodulatory effect is markedly dose-dependent.

Early studies demonstrated that increasing radiation doses were associated with enhanced ICD-related DAMP release. For instance, Golden et al. observed a dose-dependent increase in CALR exposure, ATP secretion and HMGB1 release when radiation doses were escalated from 2 to 20 Gy in vitro breast cancer models. However, accumulating evidence indicates that RT-induced ICD does not follow a linear dose–response pattern. Rather, RT alone generally induces limited ICD, with an apparent immunogenic dose window beyond which further dose escalation fails to enhance, or may even suppress, immunogenic signalling [63]. In NSCLC models, such as A549 cells, fractionated regimens (e.g. 8 Gy × 3 or 4 Gy × 2) have been shown to robustly induce ICD markers, an effect that can be further potentiated by radiosensitisers, including Bio-MnO₂ nanoparticles or ZnO-Au@mSiO₂ constructs [15, 64].

In clinical practice, patients with early-stage, inoperable NSCLC are frequently treated with high-dose stereotactic body radiotherapy (SBRT). A retrospective analysis by Koshy et al. demonstrated superior 3-year OS in patients receiving a biologically effective dose exceeding 150 Gy compared with lower-dose regimens (55% vs. 46%), although the maximum tolerated dose was not defined in that study [65]. Subsequently, Bezjak et al. established 12.0 Gy per fraction as the maximum tolerated SBRT dose, with a relatively low incidence of dose-limiting toxicity (7.2%; 95% CI: 2.8–14.5%). Comparable 2-year local control, OS, and PFS rates were observed with fraction doses of 11.5 or 12.0 Gy [66]. While SBRT achieves excellent tumour control primarily through direct cytotoxicity, its capacity to optimally activate systemic antitumour immunity may be limited, underscoring a potential divergence between regimens designed for maximal local control and those favouring ICD.

Notably, RT exerts dual regulatory effects on the TME. While inducing ICD, it can also upregulate immune checkpoint molecules such as PD-L1, thereby promoting immunosuppression. Preclinical studies confirm that specific doses of RT increase PD-L1 expression on tumour cells, DCs and macrophages, and impair CD8⁺ T-cell function within the TME [67]. There is also clinical support for this, as NSCLC patients with a history of RT show a significantly higher PD-L1 positivity rate (tumour cell cutoff ≥25%) than those without (43.7% vs. 37.1%) [68]. This provides a rationale for combining RT with ICIs, yet clinical outcomes vary (see Table 2 for more clinical data). For example, the phase II DOLPHIN trial reported favourable survival benefits (12-month PFS: 72.1%; median PFS: 25.6 months; ORR: 90.9%) with 60 Gy of RT plus durvalumab [69]. Conversely, a retrospective cohort study by Mander et al. found that palliative RT (e.g. 8 Gy × 1 or 20 Gy × 5) combined with pembrolizumab resulted in a significantly decreased PFS compared to no RT (HR = 6.254) [70]. This discrepancy likely stems from differences in RT dose and fractionation, highlighting the importance of optimising these parameters for successful combination therapy.

**Fig. 2:**
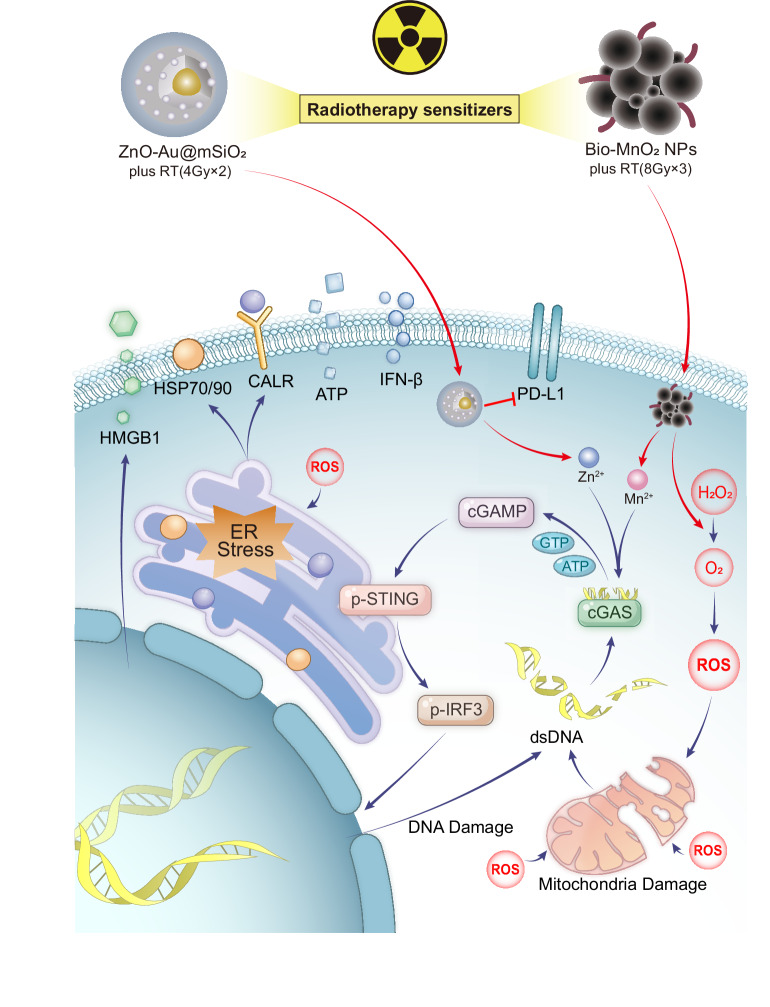
Radiotherapy sensitisers induce ICD by activating the cGAS-STING pathway. Bio-MnO₂ NPs, when combined with low-dose RT, act as radiosensitisers by promoting ROS generation and releasing Mn²⁺ to activate cGAS-STING. Similarly, ZnO-Au@mSiO₂ nanoparticles synergise with low-dose RT to trigger ICD, inhibit PD-1/PD-L1, and activate cGAS-STING.

RT-induced immunosuppression extends beyond PD-L1 upregulation to include suppression of the cGAS-STING pathway. Vanpouille-Box et al. discovered that radiation doses exceeding 12–18 Gy per fraction stimulate the production of the DNA exonuclease Trex1, which breaks down cytosolic DNA. This inhibits cGAS-STING activity and IFN-β production [71], suggesting that high-dose RT may hinder immune activation driven by ICD. Notably, Xue et al. confirmed that cGAS-STING activation enhances radiosensitivity in NSCLC [72]. In this context, nanomaterial-based strategies designed to potentiate cGAS–STING signalling represent a promising approach to optimise RT-induced ICD. For instance, Bio-MnO₂ nanoparticles combined with low-dose RT function as radiosensitisers by enhancing ROS generation while simultaneously releasing Mn²⁺ ions that activate cGAS–STING signalling, thereby promoting cytotoxic T-lymphocyte infiltration [15]. Similarly, ZnO-Au@mSiO₂ nanoparticles synergise with low-dose RT to trigger ICD, inhibit PD-1/PD-L1, and activate cGAS-STING, collectively reversing T-cell exhaustion [64]. Together, these findings suggest combining optimised (preferably moderate-to-low) RT doses with nanotechnology to activate the cGAS-STING pathway is a promising approach for enhancing immunotherapeutic efficacy (Fig. 2).

In summary, RT can induce ICD and initiate systemic antitumour immunity, however, its immunogenic potential is highly dependent on radiation dose and fractionation. While moderate RT doses promote DAMPs release and cGAS–STING–mediated immune activation, excessive doses may instead enhance immunosuppressive signalling through PD-L1 upregulation and Trex1-mediated inhibition of innate immune sensing. Therefore, optimising RT parameters, rather than simple dose escalation, is critical for maximising ICD induction and achieving effective synergy with immunotherapeutic strategies.

### Molecular targeted therapy-induced ICD

**Fig. 3:**
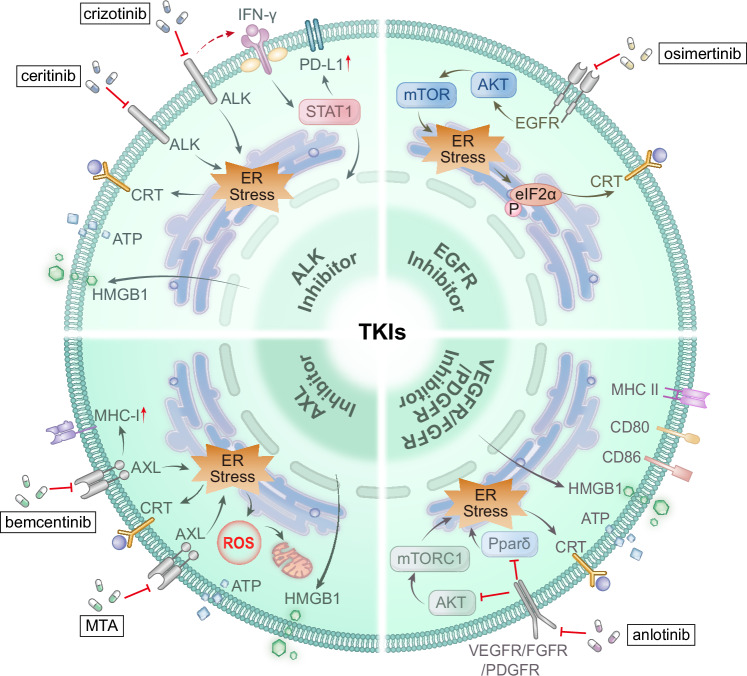
The commonalities and differences of TKIs induced ICD mechanisms. ALK inhibitors (e.g. crizotinib and ceritinib), EGFR inhibitor (osimertinib), AXL inhibitors (bemcentinib and marsdenia tenacissima extract), and VEGFR inhibitor (anlotinib) could trigger the three hallmarks of canonical ICD: CALR exposure, ATP release and HMGB1 secretion. However, the mechanistic emphasis varies. Crizotinib-induced ICD relies on the IFN-γ pathway and upregulates PD-L1. Bemcentinib modestly upregulates MHC-I expression. The marsdenia tenacissima extract induces ER stress via AXL phosphorylation inhibition, leading to mitochondrial depolarisation and ROS accumulation. Anlotinib inhibites AKT/mTORC1 and Pparδ signalling during ICD.

Recent studies have revealed that various molecular targeted agents, such as protein tyrosine kinase inhibitors (TKIs), exert antitumour effects in NSCLC, which are associated with the induction of ICD-related immunogenic signalling, and their molecular mechanisms share common features but also exhibit distinct differences. Preclinical studies, primarily based on NSCLC cell lines and murine models, suggest that ALK inhibitors (e.g. crizotinib and ceritinib), EGFR inhibitors (osimertinib), AXL inhibitors (bemcentinib and marsdenia tenacissima extract), and vascular endothelial growth factor receptor inhibitors (anlotinib) have been shown to induce key features of ICD, including CALR exposure, ATP release and HMGB1 secretion. This represents a convergent immunogenic signalling axis contributing to antitumour immune activation [17, 73–77]. However, the mechanistic emphasis varies. Crizotinib-induced ICD relies on T lymphocytes and the IFN-γ pathway, while also upregulating PD-L1 and strongly sensitising NSCLC to immunotherapy with PD-1 antibodies [73]. Osimertinib may increase plasma-soluble CALR levels in patients by upregulating the phosphorylation of eIF2α [17]. Bemcentinib has been reported to exert only modest effect on MHC-I expression [75]. In preclinical models the marsdenia tenacissima extract induces ER stress by inhibiting AXL phosphorylation, leading to mitochondrial depolarisation and ROS accumulation [76]. Anlotinib inhibits AKT/mTORC1 and Pparδ signalling to block M2 polarisation of tumour-associated macrophages during ICD [77] (Fig. 3).

In parallel with their ICD-associated immunomodulatory properties, these TKIs have demonstrated substantial clinical efficacy across multiple stages of NSCLC (see Table 2 for more clinical data). In patients with advanced ALK-positive NSCLC, crizotinib significantly prolonged PFS (HR = 0.402) and improved the ORR (87.5% vs. 45.6%) compared to chemotherapy [78]. Ceritinib has been shown to provide superior OS (HR = 0.59) and PFS (median 13.8 vs. 8.3 months) when used as initial therapy, compared to crizotinib [79]. Furthermore, it yields a better PFS than chemotherapy (median 5.4 vs. 1.6 months, HR = 0.49) in patients who have progressed following prior treatment with crizotinib [80]. For EGFR-mutant NSCLC, osimertinib has been shown to be effective at some stages of treatment: it achieved an ORR of 52% in the neoadjuvant setting [81]; when used as adjuvant therapy, it increased the 4-year disease-free survival rate to 70% (compared to 29% in the control group) and reduced the risk of central nervous system recurrence by 76% (HR = 0.24) in patients with stage IB-IIIA disease [82]; and when used as maintenance therapy for unresectable stage III NSCLC, it increased median PFS to 39.1 months (HR = 0.16) [83]. In AXL-positive advanced NSCLC, bemcentinib combined with DOC resulted in a median PFS of 3.1 months (95% CI: 1.6–5.7) and a median OS of 12.3 months (95% CI: 4.9–16.9) [84], while a marsdenia tenacissima extract-based injection plus chemotherapy improved effective rate (RR = 1.32, 95% CI: 1.14–1.54) and quality of life improvement rate (RR = 2.04, 95% CI: 1.69–2.47) [85]. Furthermore, the combination of anlotinib and a PD-1 inhibitor in patients who had progressed following prior immunotherapy treatment was associated with a disease control rate of 82.8%, a median PFS of 5.0 months (90% CI: 4.2–7.3), and a median OS of 15.1 months (90% CI: 4.2–7.3) [86]. Taken together, these data establish the clinical efficacy of TKIs in NSCLC and suggest that immune modulation involving ICD-associated mechanisms, may contribute to therapeutic outcomes. However, direct clinical evidence linking ICD induction to patient benefit remains limited, underscoring the need for biomarker-driven validation.

In summary, molecular targeted agents, including TKIs, polo-like kinase inhibitors [87], and serine/threonine kinase activators [88] (see Table 3 for a list of polo-like kinase inhibitors and serine/threonine kinase activators that could induce ICD in NSCLC), could reshape the tumour immune microenvironment by inducing ICD and synergising with ICIs. Future efforts should focus on elucidating the specific molecular pathways of ICD induction and validating efficacy through optimised combination strategies and prospective clinical trials. This will advance the precise application of targeted therapies in NSCLC treatment.

### Photodynamic therapy (PDT)-induced ICD

The induction of ICD by PDT relies on the preferential accumulation of photosensitisers (PSs) in tumour tissues, intersystem crossing-mediated singlet/triplet excitation, and subsequent generation of Type II ROS (primarily singlet oxygen, ¹O₂) via energy transfer, or Type I ROS (e.g. superoxide anion O₂•^−^, hydrogen peroxide H₂O₂, hydroxyl radical •OH) via electron transfer [89]. These ROS induce tumour cell death through both direct cytotoxicity and vascular damage, while also initiating intracellular stress responses that are critical for ICD induction. [90].

**Fig. 4:**
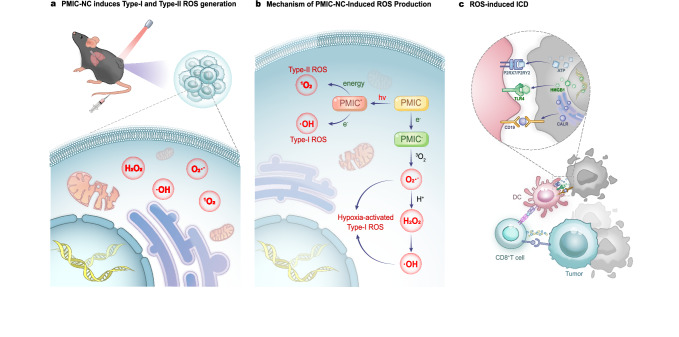
Mechanism by which the mitochondria-targeted ROS overproducer PMIC-NC induces ICD. **a** PMIC-NC induces the production of both Type I (O₂•^−^, H₂O₂, •OH) and Type II (¹O₂) ROS within the TME. **b** PMIC-NC elicites a hypoxia-enhanced Type I ROS burst via proton transients and generates Type I/II ROS through electron or energy transfer under NIR irradiation. This triggers a potent ICD effect. **c** Exposure to ROS triggers tumour cells to release ATP and HMGB1, and to translocate CALR to the cell surface. These DAMPs are recognised and taken up by DCs, which then present tumour antigens to CD4⁺ and CD8⁺ T cells. Activated CD8⁺ T cells then go on to mediate potent tumour cell killing.

The immunogenic profile of PDT-induced cell death is strongly influenced by the subcellular localisation of PSs, particularly those targeting the ER or mitochondria. Valančiūtė et al. demonstrated that methylene blue-PDT (MB-PDT) triggers ICD in NSCLC by inducing ER stress, promoting CALR membrane translocation, and elevating intercellular cell adhesion molecule-1 expression. MB-PDT inhibits NSCLC proliferation, downregulates Bcl-2 and activates Caspase 3/7. It also enhances CD8⁺ T cell-mediated tumour lysis via granzyme B in cell lines and organoids [91]. Our research group has developed a perylene monoamide-based, mitochondrial-targeted ROS supergenerator, PMIC-NC. This supergenerator not only induces hypoxia-enhanced Type I ROS bursts with the aid of proton transients but also triggers the generation of type I/II ROS through electron or energy transfer under near-infra-red radiation (NIR) irradiation, releasing ATP and HMGB1 with exposing CALR, eliciting a robust ICD effect (Fig. 4). Its mitochondrial/lung-specific localisation increases therapeutic efficacy. By integrating NIR-PDT with hypoxia-responsive chemotherapy and ICD induction, this platform enables immunogenic photochemical therapy in preclinical models of primary, distant, and metastatic tumours [16].

Novel PS systems (e.g. nano drug delivery systems and metal-based PSs) optimise NSCLC therapy. The ICG@MnO₂@Exo-anti-PD-L1 nanoplatform developed by Guo et al. reversed immunosuppression via PD-L1 blockade and generated O₂ by catalysing H₂O₂ decomposition to release Mn²⁺ and activate macrophage STING. It also triggered NF-*κ*B p65-mediated M1 polarisation and pro-inflammatory cytokine secretion alongside HMGB1 release to induce ICD in LLC cells [92]. Ai et al.’s NIR-activatable BPQDs@Lipo-YSA targeted NSCLC by inducing the PRKN/AKT1 pathway-mediated mitophagy process. Under NIR, it triggered potent ICD (CALR exposure and HMGB1 release), enhancing in vitro cytotoxicity and inhibiting in vivo tumour growth [93]. Metal-based PSs, such as specific iridium (III) complexes, have been shown to induce potent ICD in non-NSCLC models [89, 94], whereas some metal phthalocyanines [95, 96] and certain iridium (III) complexes [97, 98] have exhibited photodynamic efficacy in NSCLC models without a confirmed link to ICD highlighting the need for rigorous ICD-specific validation. Future research should assess the balance of safety and efficacy of metal-based PSs for inducing ICD in NSCLC.

Current research on PDT-induced ICD mechanisms has shifted from the cytotoxic effects to the construction of immunoregulatory networks. Future priorities include quantifying the correlations between subcellular localisation of PSs and ICD pattern, the impact of tumour heterogeneity on ICD efficiency, and the long-term biosafety of metal-based PSs. Advances in nanotechnology and mechanistic understanding are expected to facilitate the rational design of PDT-based strategies that integrate ICD induction with immunotherapy, thereby improving the precision and translational potential of PDT in NSCLC.

### Other emerging induction strategies and regulatory factors

Beyond conventional ICD-inducing therapies, emerging modalities such as oncolytic virotherapy and tumour-treating fields have been reported to induce ICD-associated features in preclinical NSCLC models, including DAMPs exposure ect (see Table 3) [99–103]. Recent studies further highlight the role of the gut microbiota in shaping ICD-associated immune responses through the gut–lung axis. Specific beneficial bacteria (e.g. Lactobacillus rhamnosus, Akkermansia muciniphila, Turicibacter and Peptococcus) can enhance antitumour immunity by promoting DCs maturation, T-cell activation and effector cell infiltration, thereby facilitating immune priming downstream of ICD. Such effects may reshape the tumour immune microenvironment to favour ICD-associated immune responses. Conversely, other gut microbiota (e.g. Enterorhabdus and Desulfovibrio) may promote an immunosuppressive microenvironment by increasing myeloid-derived suppressor cells infiltration, raising pro-tumour cytokine levels and inhibiting NK cell and M1 macrophage activation. This can potentially impair ICD-triggered immune responses [104–106]. Collectively, these findings underscore the multifaceted role of the gut microbiota in modulating ICD-associated immunity and suggest that microbiota-targeted interventions may serve as a potential adjuvant strategy to enhance ICD-based therapies in NSCLC, pending further mechanistic and clinical validation.

## Conclusion and perspectives

**Fig. 5:**
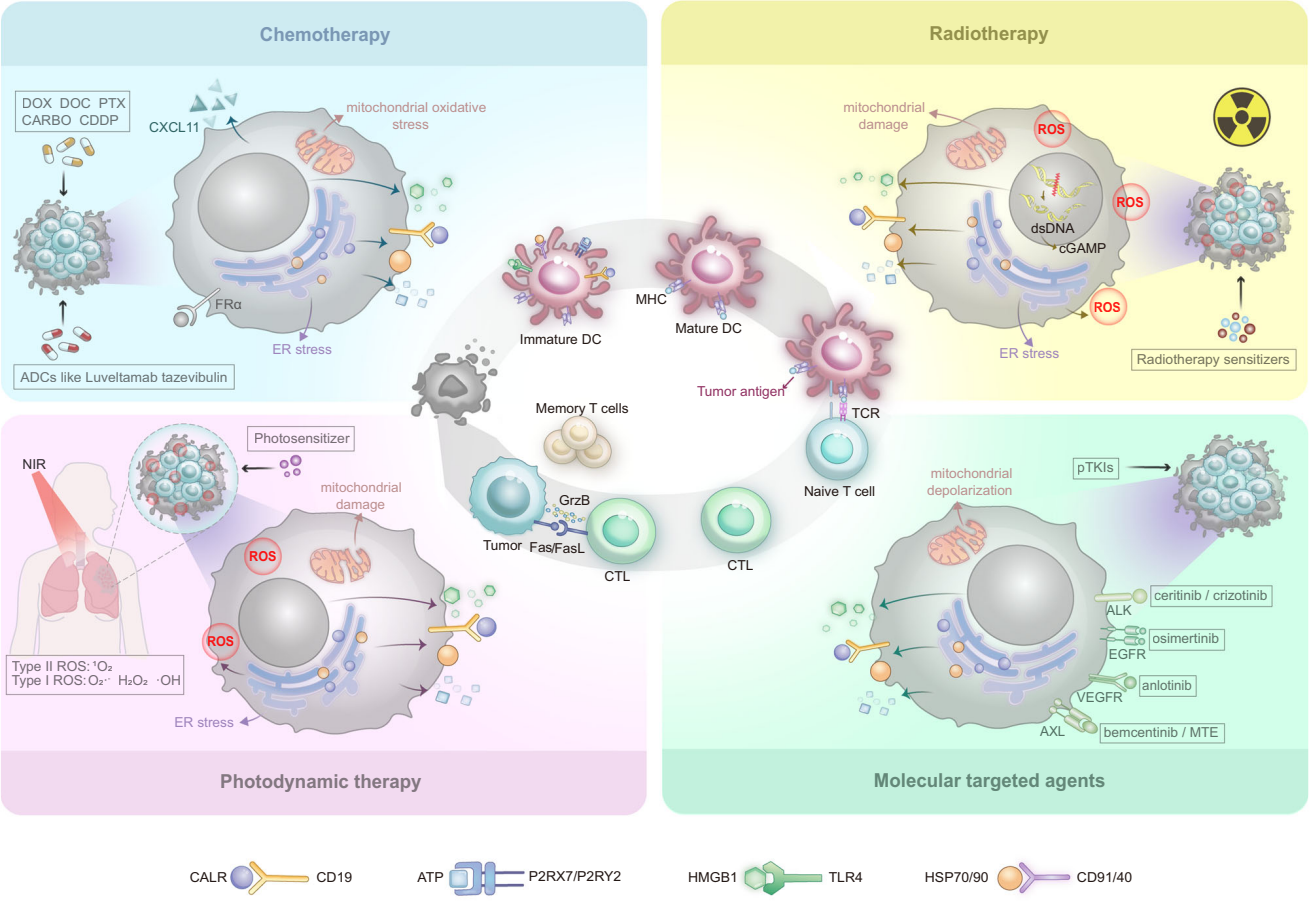
Differences and similarities in the mechanisms of induction of ICD by chemotherapy, RT, PDT, and molecular targeted therapy. Chemotherapy, RT, molecular targeted therapy, and PDT could trigger ICD and enhance the immune response through distinct mechanisms. Chemotherapy primarily induces ICD via ER stress, while RT acts through DNA damage. Molecular targeted therapies, such as TKIs, disrupt survival signalling pathways, PDT generates ROS to elicit localised stress. These therapies activate the immune response by inducing ICD, promoting DCs maturation and enhancing the killing function of CD8^+^T cells, thereby exerting antitumour effects.

In the treatment of NSCLC, inducing ICD represents an important mechanism that links direct tumour cell killing with the activation of systemic antitumour immunity. Conventional therapies, including chemotherapy, RT, molecular targeted therapy and PDT, can trigger ICD via distinct mechanisms: chemotherapy primarily through ER stress; RT through DNA damage; targeted agents by disrupting specific signalling pathways; and PDT through ROS generation (see Fig. 5 for a summary) [48, 73, 107, 108]. Combining RT and targeted therapies with ICIs can enhance ICD-associated immune priming while counteracting therapy-induced immune suppression, such as PD-L1 upregulation [67, 69, 73]. The mechanistic overlaps and differences among these modalities provide a basis for designing sequential and synergistic combination regimens.

Although extensive research related to the immunomodulatory effects of ICD has been conducted, there are several challenges to its clinical translation. Firstly, the context-dependent and heterogeneous biological effects of distinct ICD inducers across diverse tumour microenvironments require deeper mechanistic investigation in preclinical models [109]. Secondly, future clinical trials should incorporate validated ICD-related biomarkers to monitor dynamic changes in the tumour immune microenvironment of patients with NSCLC. Thirdly, personalised therapeutic strategies are required to enhance antitumour immune activation while minimising systemic toxicity.

In summary, ICD serves as a critical bridge between tumour cell death and the activation of adaptive antitumour immunity, offering a promising strategy to overcome the immunosuppressive tumour microenvironment in NSCLC and showing translational potential in clinical applications. However, its successful clinical translation requires a deeper understanding of its mechanisms, the identification of reliable biomarkers and rigorous and scientific clinical trials. Ultimately, these advances are essential for establishing a refined immunotherapeutic paradigm for NSCLC.
